# Two dimensional VOPBA reveals laminin receptor (LAMR1) interaction with dengue virus serotypes 1, 2 and 3

**DOI:** 10.1186/1743-422X-2-25

**Published:** 2005-03-25

**Authors:** Phaik Hooi Tio, Wan Wui Jong, Mary Jane Cardosa

**Affiliations:** 1 Institute of Health and Community Medicine, Universiti Malaysia Sarawak, 94300 Kota Samarahan, Sarawak, Malaysia

## Abstract

**Background:**

The search for the dengue virus receptor has generated many candidates often identified only by molecular mass. The wide host range of the viruses *in vitro *combined with multiple approaches to identifying the receptor(s) has led to the notion that many receptors or attachment proteins may be involved and that the different dengue virus serotypes may utilize different receptors on the same cells as well as on different cell types.

**Results:**

In this study we used sequential extraction of PS Clone D cell monolayers with the detergent β-octylglucopyranoside followed by sodium deoxycholate to prepare a cell membrane-rich fraction. We then used 2 dimensional (2D) gel electrophoresis to separate the membrane proteins and applied a modified virus overlay protein binding assay (VOPBA) to show that dengue virus serotypes 1, 2 and 3 all interact with the 37 kDa/67 kDa laminin receptor (LAMR1), a common non-integrin surface protein on many cell types.

**Conclusion:**

At least 3 of the 4 dengue serotypes interact with the 37 kDa/67 kDa laminin receptor, LAMR1, which may be a common player in dengue virus-cell surface interaction.

## Background

The dengue viruses have become recognized as important global pathogens causing dengue haemorrhagic fever not only in Southeast Asia but also in South and Central America and in the Caribbean.[1,2]. There are 4 closely related dengue viruses referred to as DENV-1, DENV-2, DENV-3 and DENV-4[3]. They are mosquito borne viruses with a single stranded positive sense RNA genome around 11 kilobases in length, and are able to infect both mosquito and human hosts. A wide range of cell types from multiple species is susceptible to infection with dengue viruses *in vitro*. Numerous studies have attempted to identify the cell surface receptor or receptors utilized by the dengue viruses to gain entry into susceptible cells, but multiple approaches using different cell lines and different dengue virus strains have generated many candidate DENV interacting proteins identified in some cases only by molecular mass [4-11]. Heparan sulfates[12] and the C-type lectins DC-SIGN and L-SIGN have been shown to mediate infection by dengue viruses[13] and most recently, studies using a standard virus overlay protein binding assay (VOPBA) have suggested that in the liver cell line HepG2, different DENV serotypes utilize different cell surface molecules[14]. More specifically, mass spectrometric methods have been used to identify reactive bands using VOPBA and it has been suggested that DENV-2 interacts with GRP78[15] while DENV-1 interacts with the 37 kDa/67 kDa high affinity laminin receptor[16].

**Figure 1:**
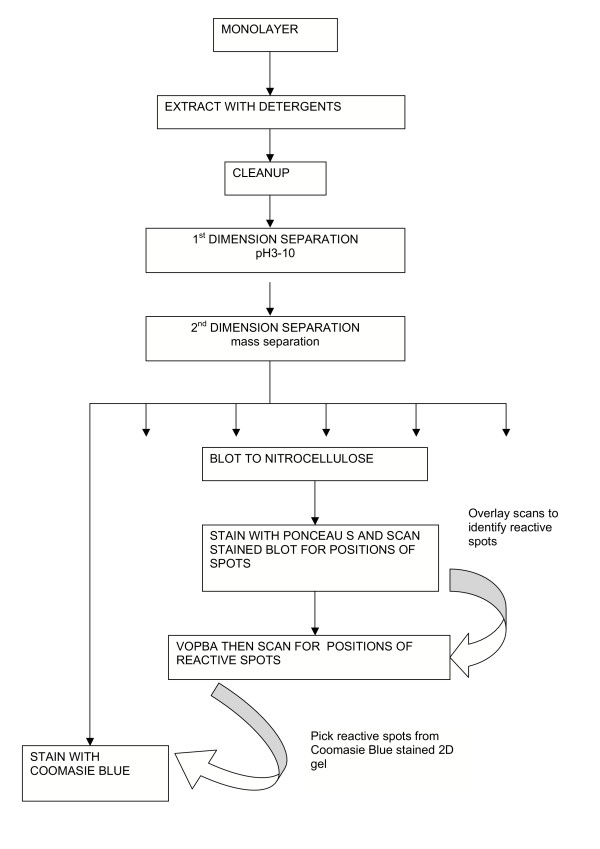
**Flowchart of experimental design. **This describes the steps in the process of identifying dengue virus reactive proteins by 2D VOPBA to facilitate picking the relevant spots from a gel run under identical conditions and at the same time as those which were used for electrotransfer to nictrocellulose membranes.

In a standard VOPBA, complex protein preparations are separated according to molecular mass in a single dimension and transferred to membranes to be probed with virus antigen[17,18]. When complex mixtures are used there are often many co-migrating proteins that cannot be adequately resolved for accurate interpretation of mass spectrometric data when single dimension separations are used. We have used two dimensional (2D) gel electrophoresis[19] for separation of cell membrane preparations. After electrotransfer of 2D gel-separated proteins to nitrocellulose membranes, we probed the membranes with various dengue virus antigen preparations. This 2D VOPBA approach has facilitated the separation of multiple proteins of similar molecular mass along a pH gradient. Reactive spots recovered using this approach were identified on companion 2D gels using matrix assisted laser desorption ionization-time of flight mass spectrometry (MALDI TOF MS)[20]. A schematic diagram of our experimental design is shown in figure 1. To the best of our knowledge, this is the first description of the use of 2D VOPBA for identification of proteins interacting with flaviviruses.

## Results

### Identification of reference protein spots in detergent extracts of PS Clone D cell monolayer

**Figure 2:**
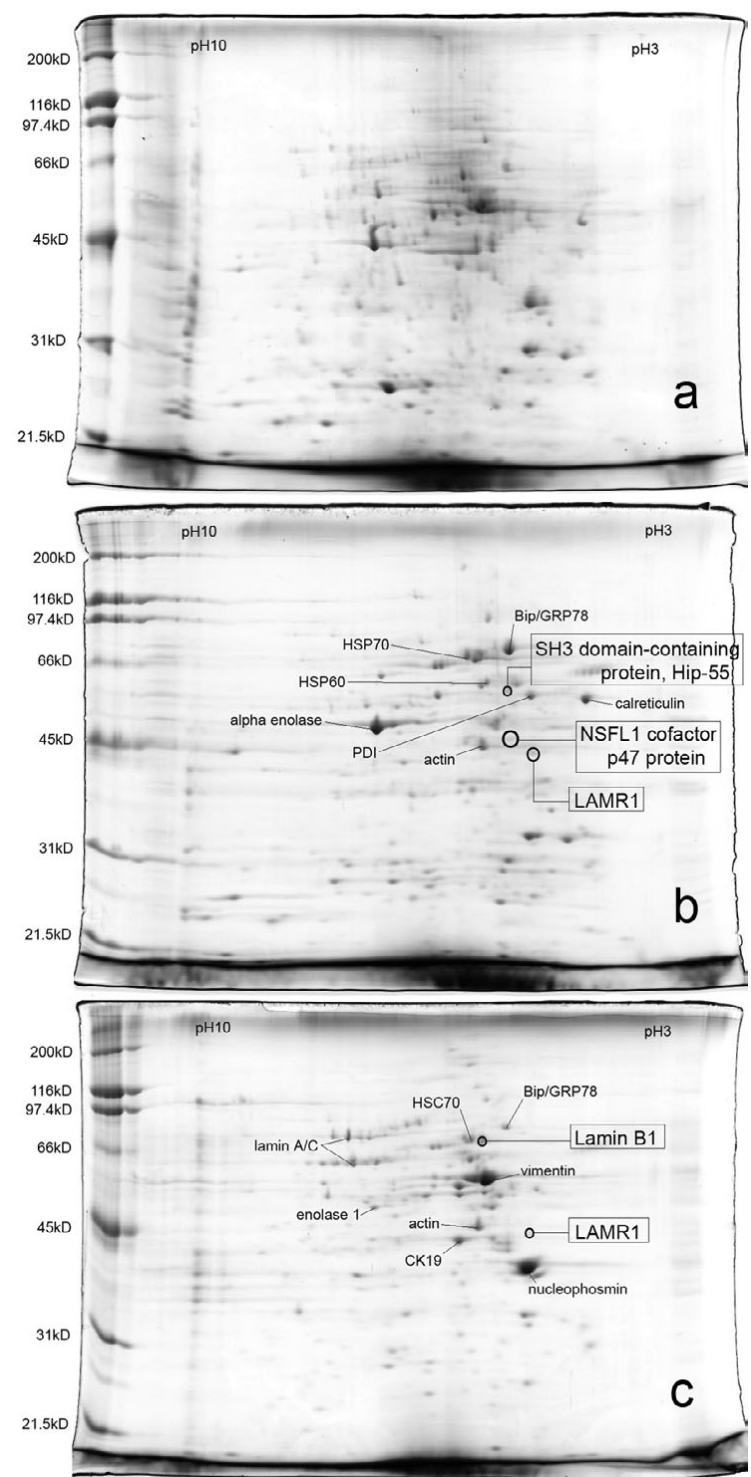
**2D gel electrophoresis image of detergent extracts from PS Clone D cell monolayer. **The first dimension was run on linear 7 cm IPG strip, pH 3-10. The second dimension was 10% SDS PAGE. The gel was stained with coomasie brilliant blue and spots were picked and subjected to in-gel trypsin digestion. The major spots identified by MALDI TOF are labelled. Circled spots were reactive in the 2D VOPBA. (a) NaDOC-extract (b) βOG-extract (c) NaDOC-extract after removal of βOG-soluble proteins.

The proteomic profile of 1% sodium deoxycholate (NaDOC) soluble proteins from PS Clone D cell monolayers is shown in figure 2a. Numerous spots were seen and it was necessary to attempt to fractionate the components of the cell monolayer by sequential treatment with 1% β octyl-glucopyranoside (βOG) followed by 1% NaDOC. Beta octyl-glucopyranoside soluble proteins and post-βOG residual NaDOC-extracted proteins were resolved separately on 2D gels. Major protein spots were picked and subjected to in-gel trypsin digestion followed by MALDI-TOF MS. It was found that the βOG extract contained both cytoplasmic as well as some membrane proteins while the post-βOG residual NaDOC extract contained mainly cytoskeletal and nucleus associated proteins. Figures 2b and 2c show some of the major spots identified and used as identifying features when comparing overlays of Ponceau S stained blots with VOPBA developed blots. In figure 2b showing separation of βOG extracts, BiP or GRP78 was prominent as was calreticulin, alpha enolase, HSP70, PDI and actin. Circled spots were reactive in the 2D VOPBAs described later and are shown here to provide the protein landscape in which these reactive proteins exist. In figure 2c, showing separation of βOG-insoluble NaDOC-extracted proteins, BiP/GRP78 was less prominent but vimentin and nucleophosmin were highly prominent. Again the circled spots mark the positions of VOPBA-reactive spots, showing the location of these very sparse proteins in the landscape of much more abundant protein spots.

### 2D VOPBA of β octyl-glucopyranoside extract

**Figure 3:**
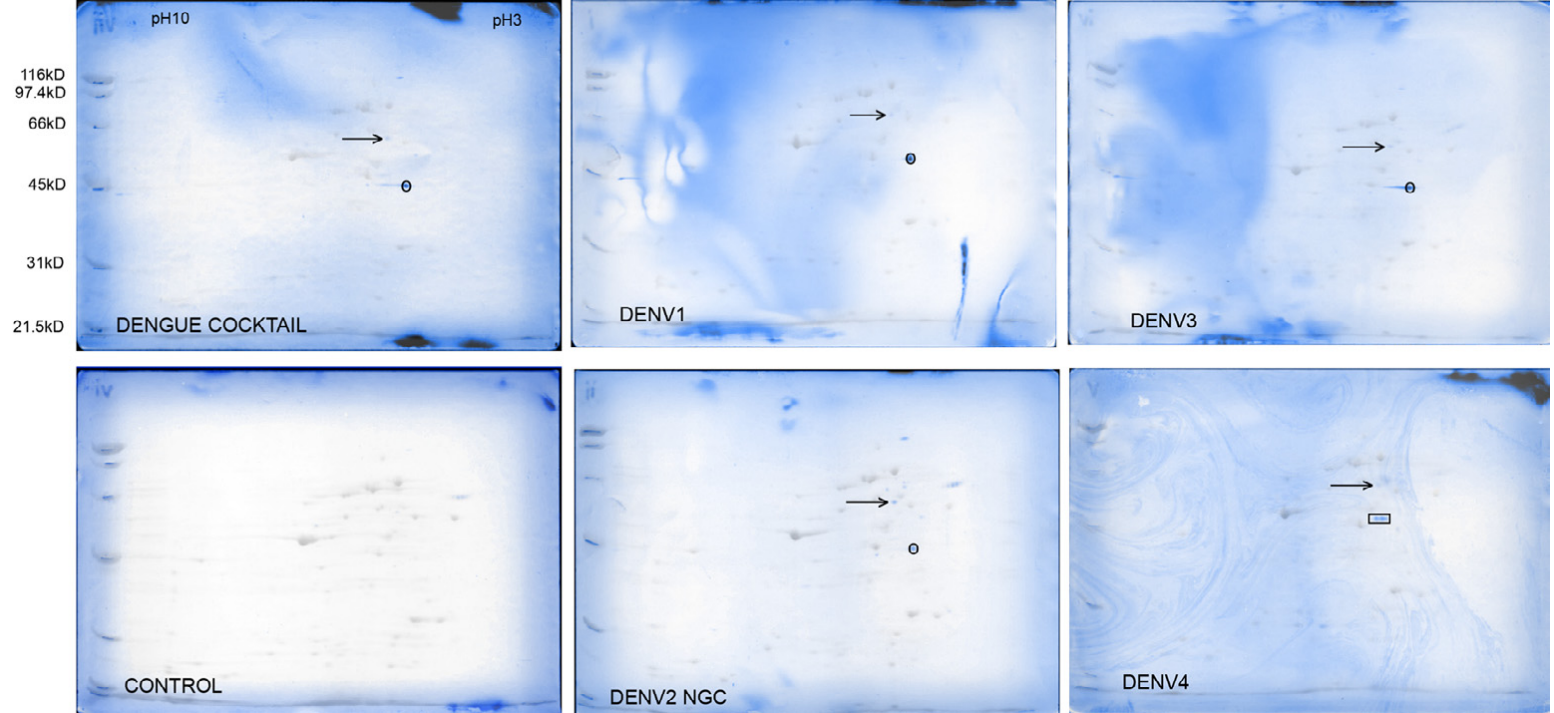
**2D VOPBA of β octyl-glucopyranoside soluble proteins from PS Clone D cell monolayer. **The antigen preparation used in the VOPBA staining of the 2D blots is labelled on the bottom left of each blot. Two images are superimposed in each panel. The Ponceau S scan of each blot is shown in greyscale and shows the universe of spots transferred to the blot. The spots reactive in the VOPBA analysis are shown in blue. Spots marked with an arrow were identified as Hip-55, those marked with a circle were identified as LAMR1 and the pair of spots in the DENV4 blot marked with a rectangle were identified as p47 protein (NSFL1 cofactor).

2D gel blots of βOG extracted PS Clone D cells were probed with a cocktail of antigens prepared from 4 different dengue virus serotypes grown in C6/36 mosquito cells. Bound envelope protein (E) was detected by using the monoclonal antibody 4G2, a flavivirus-reactive anti-E antibody[21]. Replicate blots were also probed with individual dengue serotypes separately. Antigens prepared from uninfected mosquito cells were used as negative controls in order to identify non-dengue specific interactions present in all blots. These blots are shown in figure 3. In the 2D VOPBA blots probed with a dengue virus antigen or cocktail, 2 reactive spots were seen. The blots probed with dengue cocktail, DENV-1, DENV-2 and DENV-3 had identical reactivities. The major spot that was clearly reactive was around 45 kD in molecular mass with a pI of 4–5. A weaker, less obvious reaction was seen with a spot at 50–60 kD and a slightly higher pI of around 5. MALDI-TOF MS-generated peptide mass fingerprints identified these spots as 37 kDa/67 kDa laminin binding protein or laminin receptor (LAMR1) and an SH3 domain-containing protein, Hip 55, respectively. The blot probed with DENV-4 did not show any reactivity with LAMR1 but there was a weak reactivity with Hip 55. Furthermore, a clearly reactive pair of spots identified as p47 protein (a cofactor of NSFL1/p97) was seen in the DENV-4 VOPBA.

### 2D VOPBA of sodium deoxycholate extract of cells previously extracted with β octyl-glucopyranoside

**Figure 4:**
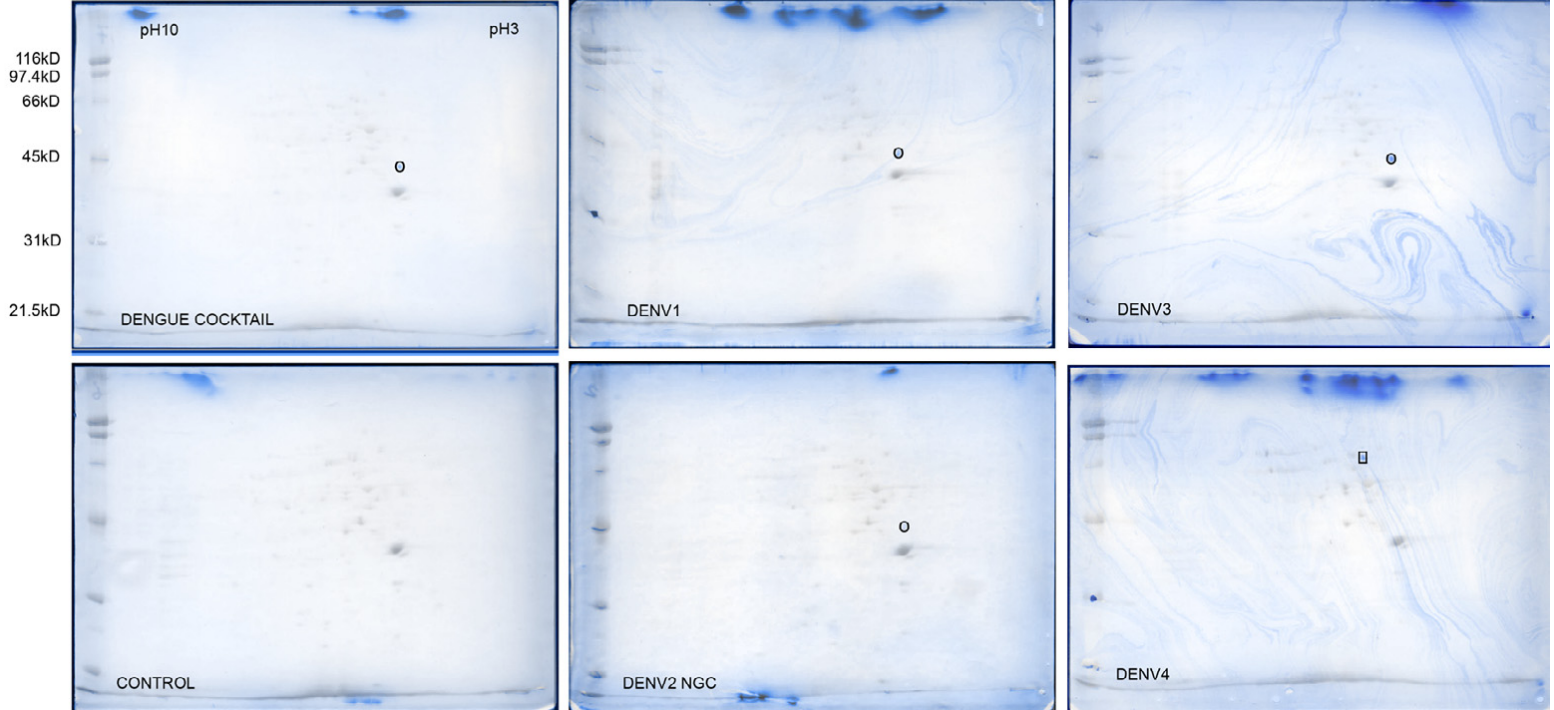
**2D VOPBA of sodium deoxycholate soluble proteins after β octyl-glucopyranoside extraction of PS Clone D cell monolayer. **The antigen preparation used in the VOPBA staining of the 2D blots is labelled on the bottom left of each blot. Two images are superimposed in each panel. The Ponceau S scan of each blot is shown in greyscale and shows the universe of spots transferred to the blot. The spots reactive in the VOPBA analysis are shown in blue. Spots marked with a circle were identified as LAMR1 and the spot in the DENV4 blot marked with a rectangle was identified as lamin B1.

Residual protein from PS Clone D cells treated with βOG was further treated with NaDOC and the resulting extract was used to prepare 2D gel blots. VOPBAs were performed as above and figure 4 shows the individual VOPBA blots. As with the βOG-extract, LAMR1 was found to be reactive in VOPBA blots probed with dengue cocktail, DENV-1, DENV-2 and DENV-3, but not DENV-4. Instead, the DENV-4-probed blot showed a clear reaction with a protein of higher molecular mass and higher pI than LAMR1. This protein was identified to be lamin B1, a nuclear membrane protein.

### MALDI TOF MS

Peptide mass fingerprints were obtained using Voyager DE STR (Applied Biosystems, Foster City, CA, USA) and mass lists were submitted for search against the NCBI protein database (National Institutes of Health, Bethesda, MD, USA) using the MASCOT search engine[22]. Spectra and their corresponding mass-lists generated from analysis of the tryptic digests are provided as additional files 1,2,3,4,5.

### Immunoblot confirmation of LAMR1 and lamin B1 spots on 2D gel blots

**Figure 5:**
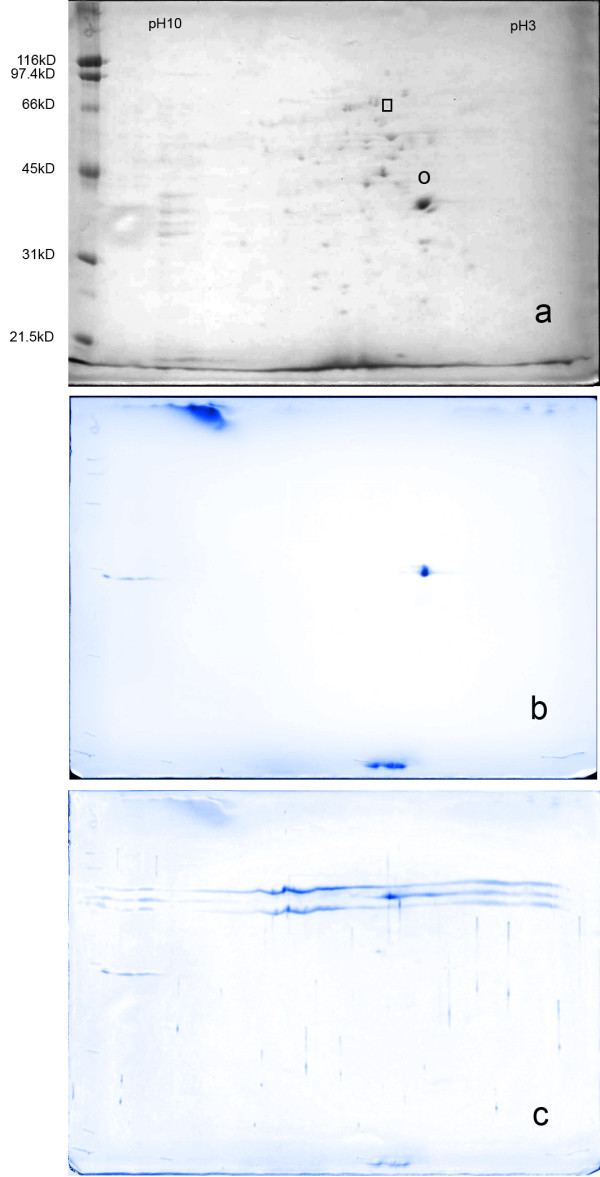
**2D gel immunoblots probed with LAMR1 and lamin B1 specific antisera. **Two dimensional separation of βOG-insoluble NaDOC extracts transferred to nitrocellulose and probed with specific antisera shows specific staining of the MALDI TOF MS identified spots. Panel (a) shows a Ponceau S stained blot of a 2D gel prior to probing with specific antibody, (b) shows a blot probed with LAMR1 specific antisera and (c) shows a blot probed with lamin B1 specific antisera. In panel (a) the circle marks the position of LAMR1 and the rectangular box marks the position of lamin B1.

The NaDOC soluble proteins from βOG extracted PS Clone D cells were used to prepare 2D gel blots and these were probed with antibodies specific for LAMR1 and lamin B1. Figure 5a shows a Ponceau S stained blot and the dengue virus antigen reactive spots are circled. The locations of specific staining by antibodies to LAMR1 and lamin B1 on the 2D blots were confirmed to be in the positions of the spots identified by MALDI TOF MS as LAMR1 and lamin B1 (figures 5b and 5c).

### LAMR1 is expressed on the surface of PS Clone D cells

**Figure 6:**
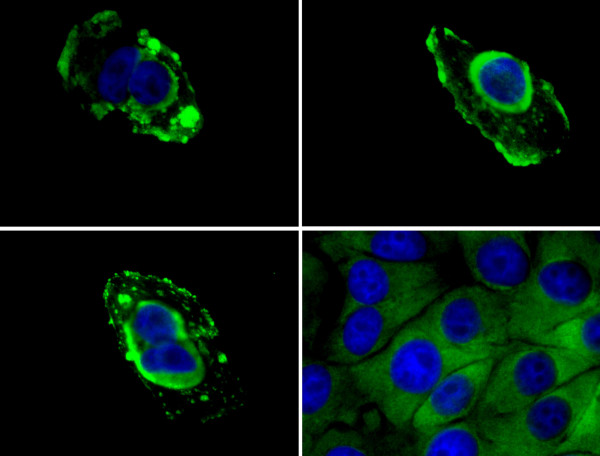
**LAMR1 is expressed on the surface of PS Clone D cells. **The top 2 panels and the bottom left panel show individual cells with patches of bright staining of LAMR1 on the surface of paraformaldehyde fixed cells. When contact inhibition occurred as shown in the bottom right panel, LAMR1 was more evenly distributed and the dense patches of LAMR1 were no longer present.

When PS Clone D cells were fixed with paraformaldehyde LAMR1 staining was seen on the surface of the cells. In keeping with observations of de Hoog and coworkers[23] we found that single cells at the edges of the monolayer were consistently displaying brightly fluorescent patches on the cell surfaces showing high levels of expression of LAMR1. In the centres of the monolayers where cells were contact inhibited, LAMR1 staining was more muted and more evenly distributed over the cells (see figure 6).

## Discussion

The investigation of early events in the infection of susceptible cells by dengue viruses is important in the quest to understand the ability of this group of mosquito borne viruses to infect both insect and mammalian cells, yet appear to have a restricted tissue tropism in the human host. Determination of the nature of the early interactions of the infecting viruses with molecules on the surface of susceptible cells provides for the possibility that this understanding can lead to the development of therapeutic agents that can be used to inhibit virus infection. The laminin receptor has already been described as a receptor for DENV-1 by single dimension VOPBA followed by MS/MS[16]. The results from the MASCOT[22] search showed multiple hits in this particular study, indicating several possibilities, including ATP synthase β chain, β actin and the laminin receptor, but the authors selected the lower scoring laminin receptor for further investigation. This was a reasonable choice since this molecule has previously been identified as a receptor for the alphaviruses Sindbis virus[24] and Venezuelan equine encephalitis virus (VEEV)[25] and the flavivirus tick-borne encephalitis virus (TBEV)[26].

In our study, 2D VOPBA was used to eliminate the problem of co-migrating bands in a single dimension and the mass list generated from trypsin digestion of the most prominent reactive spot turned up numerous hits unambiguously listing the same protein, variously known as protein 40 kD, laminin-binding protein, 34/67 kDa laminin receptor, laminin receptor 1, LAMR1, Lamr1 protein, 67 kDa laminin receptor, 40S ribosomal protein SA, and so on. Contrary to earlier suggestions that LAMR1 is a DENV-1 specific receptor[14,16], in our hands, DENV-1, DENV-2 and DENV-3 were all shown to interact with the same molecule LAMR1 although DENV-4 did not. It is thus likely that in the PS Clone D cells we have studied, at least 3 of the 4 different dengue serotypes utilize the same surface protein to gain entry into the cells. In our study we also did not find any evidence of DENV-2 binding to BiP/GRP78 as has been shown using single dimension VOPBA[15].

LAMR1 is a non-integrin receptor interacting with the extracellular matrix. It is generally accepted that the 37 kDa form is the precursor to the 67 kDa form although it is still not clear how this transition occurs. We have used a commercially available rabbit polyclonal antibody raised against a recombinant protein corresponding to amino acids 110–250 of human LAMR1 to show that this protein can be found in patches on the surface of PS clone D cells which are not contact inhibited *in vitro*. This distribution of LAMR1 is consistent with the findings of Donaldson et al[27], and it is thus possible that LAMR1 may be utilized as a receptor by the dengue viruses. Other groups have already shown that LAMR1 is a functional receptor for Sindbis virus, VEEV, TBEV and DENV1[16,24-26]. We have not included functional studies in this present work, as this study is meant to be an exploratory study of dengue virus interacting proteins in PS Clone D cells using 2D VOPBA as an interrogating tool. There is no doubt that the treatment of the cell extracts limits the ability of this method to identify interactions dependent upon conformational structures, nevertheless, we managed to identify LAMR1 confirming previous observations by other investigators[27]. We have further shown that LAMR1 interaction is not limited to DENV-1 alone, but that DENV-2 and DENV-3 also interact with LAMR1 and that this may be a common receptor for dengue virus entry into cells.

Many integrins have been shown to function as receptors for different viruses, for example the α6 integrins mediate human papillomavirus entry[28], β3 integrins mediates cell entry by hantaviruses.[29], α2β1 integrin is a receptor for human echovirus 1.[30,31], α5β1 integrin binds human parvovirus B19[32], α2β1 and αxβ2 mediate rotavirus infection[33] and αvβ3 is the receptor for the flavivirus West Nile virus (WNV).[34]. Many viruses are now known to infect cells through a multistep process involving binding to the cell surface followed by internalization, often through interacting with more than one surface molecule. Outside-in binding of integrins leads also to signal transduction, and this functional activation has been shown to be necessary for internalization as in the example of human parvovirus B19. In the case of adenoviruses, integrin clustering due to receptor binding initiates the signalling events required for internalization[35]. The finding that DENV-1, DENV-2 and DENV-3 interact with the laminin receptor is thus consistent with this growing body of work describing the utilization by viruses of extracellular matrix protein receptors for gaining entry into cells.

In this study we have also identified dengue virus envelope protein interaction with an actin binding protein Hip55, which has been shown to be involved in endocytosis, vesicular transport and signal transduction[36]. Hip55 has also been shown to interact with CD2v protein of African swine fever virus (ASFV) and colocalizes to areas surrounding perinuclear virus factories in ASFV infected cells[37]. The role of Hip55 in the life cycle of dengue virus should be further investigated.

In our preparations of dengue virus antigens, DENV-4 antigen had the weakest reactivity in ELISA (data not shown) suggesting a lower titre of antigen than the other 3 serotypes, but the DENV-4 2D VOPBA did show a different reactivity pattern, picking out lamin B1 instead of LAMR1. Lamin B1 is not a plasma membrane protein but is part of the nuclear membrane[38,39] and is thus unlikely to be an alternative receptor for DENV-4. The significance of the reaction of DENV-4 envelope protein with lamin B1 is unclear and will be the subject of further studies. It is also interesting that DENV-4 envelope also reacted with another protein involved in vesicle transport and target membrane fusion, the p47 protein cofactor of NSFL1/p97[40]. The similarity of function of the p47 protein with that of Hip 55, which is reactive with all 4 dengue serotypes, suggests that DENV-4 may use a different pathway than DENV-1, DENV-2 and DENV-3 in the course of infection of a particular cell.

## Conclusion

Two-dimensional VOPBA was used to identify cell membrane proteins interacting with dengue virus envelope protein. This approach identified several interactors including LAMR1, a non-integrin laminin binding protein, which has previously been suggested as a receptor for DENV-1 but not other dengue virus serotypes. Using more rigorous tools we have shown clearly that LAMR1 interacts not only with DENV-1 but also with DENV-2 and DENV-3. We have further shown that dengue virus envelope protein from all 4 serotypes also interacts with an actin binding protein Hip55 and that DENV-4 differs from the other three dengue virus serotypes in that it's envelope protein interacts with lamin B1 and p47 and does not interact with LAMR1.

## Methods

### Preparation of virus antigens

The 4 prototype dengue viruses were used in this study. All viruses were propagated in *Aedes albopictus *C6/36 cells grown in Leibovitz 15 media supplemented with 5% heat inactivated foetal calf serum, antibiotics and 10% tryptose phosphate broth. Antigens were prepared by inoculating C6/36 cell monolayers with the different DENV serotypes as described previously[41] and harvested when syncytium formation was extensive. Cell culture fluids were clarified by centrifugation before use. Fluids similarly prepared from mock infected C6/36 cells were used as negative antigen controls.

### Preparation of cell and membrane extracts

Just confluent flasks of the porcine kidney cell line PS Clone D were used in the preparation of detergent extracts for separation by 2D gel electrophoresis. Monolayers were washed twice with phosphate buffered saline (PBS) before subjecting to treatment with 1% β octyl-glucopyranoside (βOG) in a hypotonic buffer containing 10 mM HEPES, 1.5 mM MgCl_2_, 5 mM KCl and a protease inhibitor cocktail (Boehringer Mannheim GmbH, Germany), pH 7.5 rocking at 4°C for 1 hour. The solution was removed and designated βOG-extract.

The remaining membranes, cytoskeleton and nuclei were then washed for 1 hour at 4°C with a solution containing 2% CHAPS in the same buffer as described above. The resulting solution was discarded and the residual material solubilized by rocking at 4°C for 1 hour in the above buffer containing 1% sodium deoxycholate (NaDOC). The resulting solution was removed and designated βOG-insoluble NaDOC-extract.

All extracts were spun in a microfuge at 14,000 rpm for 10 minutes and the supernatants stored at -20°C until use.

### Sample preparation for 2D gel electrophoresis

All samples were prepared for 2D gel electrophoresis using the Ready Prep 2-D Cleanup Kit (BioRad Laboratories, Hercules, CA, USA) according to the manufacturer's instructions.

### 2D gel electrophoresis

Protein pellets were resolubilized in IPG strip rehydration solution (8 M urea, 2% CHAPS, 40 mM DTT, 0.5% IPG buffer pH3-10, bromophenol blue) at room temperature for 30 minutes, then spun in a microfuge at 14,000 rpm for 10 min. 125 ul of the resulting supernatant was used for each IPG strip (ReadyStrips pH 3-10, 7 cm, BioRad Laboratories, Hercules, CA, USA) and rehydration was achieved at 50 uA for 15 hours at 20°C using the IPGphor IEF system (Amersham Pharmacia Biotech, Uppsala, Sweden). Subsequently IEF was carried out for 30 minutes at 500 V, 30 minutes at 1000 V and 2 to 2.5 hours at 8000 V with a step-and-hold gradient until a total of 8500 volt-hours had been achieved.

IPG strips were then washed with distilled water and then equilibrated by rocking for 20 minutes at room temperature in SDS equilibration buffer (50 mM Tris-HCl pH 8.8, 6 M urea, 30% glycerol, 2% SDS) containing 10 mg/ml DTT, allowing for at least 5 ml of buffer per strip.

Strips were then washed with distilled water and placed on the top surface of the second dimension gel which was a 10% SDS polyacrylamide gel polymerized overnight. Molecular weight markers were applied onto small pieces of chromatography paper and inserted next to each strip on the top of each gel, after which the strips and markers were sealed with 0.7% agarose in 0.125 M Tris-HCl pH 6.8. The second dimension separation of proteins by molecular mass was achieved at a constant 140 V (Mini Protean 3, BioRad Laboratories, Hercules, CA, USA).

### Electrotransfer of 2D gels to nitrocellulose

The Hoefer TE series Transfor Electrophoresis Unit (Hoefer Scientific Instruments, San Francisco, CA, USA) was used to electrotransfer proteins from 2D gels to nitrocellulose membranes at 200 mA for 1 hour in ice cold Towbin buffer (25 mM Tris, 192 mM glycine, 20% methanol). Nitrocellulose blots were then stained using Ponceau S. A record of the positions of the visible protein spots on each blot was made by scanning the Ponceau S probed blot using ImageScanner (Amersham Pharmacia Biotech, Uppsala, Sweden) and the software ImageMaster Labscan v3.00 (Amersham Biosciences, UK). After scanning, the Ponceau S was stripped by washing in water and the blots were then blocked by rocking for 1 hour in PBS containing 5% skimmed milk.

### Virus overlay protein binding assay (VOPBA)

The 2D blots were incubated overnight with rocking at room temperature with clarified antigen preparations and mock-infected controls. The blots were then washed with PBS and incubated with the anti-flavivirus monoclonal antibody 4G2 in another overnight incubation at room temperature. After washing with PBS, the blots were incubated with rabbit-anti-mouse Ig HRP (DAKO, Glostrup, Denmark) at 1:1000 dilution in 5% skimmed milk in PBS for 2 hrs at room temperature. The blots were then washed with PBS and reactive protein spots were visualized by developing with the chromogenic substrate, 4-chloro-1-naphthol/hydrogen peroxide. Reaction was stopped after 1 hr by washing with water. The membranes were scanned and compared with the Ponceau S images scanned previously using Adobe Photoshop version 5.0 LE (Adobe Systems Inc., San Jose, CA, USA).

### In-gel trypsin digestion and analysis by MALDI-TOF MS

Reactive spots seen on the blots were identified in the Ponceau S scans which had been recorded previously and the corresponding spots in the coomassie blue-stained gel were picked and stored in UHQ water in 0.5 ml microfuge tubes at 4°C. During all steps in the digestion process the buffer used was 5 mM NH_4_HCO_3_. Gel spots were first destained with 50% HPLC grade methanol. Destained spots were dehydrated with acetonitrile for 10 minutes before incubation with 10 mM DTT for 50 minutes at 55°C. This was followed by incubation with 55 mM iodoacetamide (IAA) for 30 minutes at room temperature in the dark. The spots were then washed twice with buffer for 20 minutes each time, dehydrated with acetonitrile for 10 minutes and rehydrated with buffer. Finally the gel spots were dehydrated twice with acetonitrile for 10 minutes each time and dried completely by centrifugation under vacuum (DNA Speed-Vac DNA110, Savant Instruments Inc, Farmingdale, NY, USA) for 10 minutes. Each gel spot was then reswelled in 5 ul of 12.5 ng/ul of sequencing grade trypsin (Promega, Madison, WI, USA) in 5 mM NH_4_HCO_3 _for 45 minutes on ice. Excess trypsin solution was then removed, the spots were covered in 5 ul buffer and digestion was allowed to proceed at 37°C overnight. Digests were stored at -20°C until analysed.

### Analysis by MALDI-TOF MS

For MALDI analysis, digests were thawed, spun in a microfuge at 14,000 rpm for 10 minutes. One ul of the supernatant was mixed in a 1:1 ratio with a 1:10 dilution of saturated α-cyano-4-hydroxycinnamic acid (ACCA) matrix in 0.25% trifluoroacetic acid, 50% acetonitrile, 50% water. This mixture was spotted onto MALDI target plates and spectra were acquired using Voyager-DE STR Biospectrometry workstation (Applied Biosystems, Foster City, CA, USA). Peptide mass lists were submitted for search against the NCBI database (National Institutes of Health, Bethesda, MD, USA) using the MASCOT search engine (Matrix Science, London, UK). No constraints were set for species but carbamiodomethylation of cysteine residues and possible missed-cleavages were included.

### Immunostaining of 2D gel blots

The 2D blots were incubated with polyclonal rabbit antisera against LAMR1 and Lamin B1 diluted 1:200 in PBS with 5% skimmed milk at room temperature, overnight with rocking. After extensive washing with PBS, the bound antibodies were detected with anti rabbit Ig conjugated with horseradish peroxidase, and visualized using the chromogenic substrate 4-chloro-1-naphthol/hydrogen peroxide as described above. Antisera were obtained from Santa Cruz Biotechnology (Santa Cruz, CA, USA).

### Surface staining of cells and photomicrography

Cells were resuspended at 1 × 10^5 ^cells per ml in Leibovitz 15 media containing 3% heat inactivated foetal calf serum, antibiotics and tryptose phosphate broth. Resuspended cells were delivered in 25 ul volumes to individual wells of multitest slides (Erie Scientific Co., Portsmouth, NH, USA) and allowed to adhere overnight in a moist box at 37°C. Cells were then washed in PBS and fixed with 3.7% paraformaldehye in PBS at pH 7.4 for 15 minutes followed by a shift to 2% paraformaldehyde in PBS at pH 8.5 for a further 15 minutes. After washing in PBS slides were air dried and stored at -20°C until use.

Prior to staining, slides were incubated in 50 mM ammonium chloride in PBS for 5 minutes, washed thoroughly and blocked in 1% foetal calf serum in PBS for 30 minutes. Immunofluorescence staining of the surface of cells was achieved by incubation for 1 hour with polyclonal rabbit antisera against LAMR1 at 1:25 dilution in PBS containing 1% foetal calf serum. After washing with PBS the cells were incubated with anti rabbit Ig conjugated with Alexa Fluor 488 (Molecular Probes, Eugene, OR, USA) at 1:1000 dilution for 30 minutes following by washing with PBS. DAPI was used to counterstain nuclei. Slides were viewed using an Axiovert 200 (Zeiss, Germany) with filter sets appropriate for FITC and DAPI.

Photomicrography was achieved using a cooled CCD monochrome 12 bit camera Evolution QEi and Image-Pro 5.0 software (Media Cybernetics Inc., Canada) was used for preparing fluorescence composite images with pseudocolour. Adobe Photoshop version 5.0 LE was used to compose and present the figure collage.

## Competing interests

The author(s) declare no competing interests in relation to this work.

## Authors' contributions

PHT and WWJ performed the proteomics work, MJC and PHT prepared the virology reagents and the immunofluorescence. All authors contributed to the analysis and writing of the paper.

## Supplementary Material

Additional File 1 **Spectra acquired with mass list submitted for search **Image of spectra obtained by MALDI-TOF for the spot from the 2D gel of βOG extracts identified as LAMR1. Click here for file

Additional File 2 **Spectra acquired with mass list submitted for search **Image of spectra obtained by MALDI-TOF for the spot from the 2D gel of NaDOC extracts identified as LAMR1. Click here for file

Additional File 3 **Spectra acquired with mass list submitted for search **Image of spectra obtained by MALDI-TOF for the spot identified as lamin B1. Click here for file

Additional File 4 **Spectra acquired with mass list submitted for search **Image of spectra obtained by MALDI-TOF for the spot identified as Hip 55. Click here for file

Additional File 5 **Spectra acquired with mass list submitted for search **Image of spectra obtained by MALDI-TOF for the spot identified as p47 protein. Click here for file

## References

[B1] Gibbons RV, Vaughn DW (2002). Dengue: an escalating problem. Bmj.

[B2] Gubler DJ (2004). The changing epidemiology of yellow fever and dengue, 1900 to 2003: full circle?. Comp Immunol Microbiol Infect Dis.

[B3] van Regenmortel MHV, Fauquet CM, Bishop DHL, Carsten EB, Estes MK, Lemon SM, Maniloff J, Mayo MA, McGeoch DJ, Pringle CR, Wickner RB (2000). Virus Taxonomy: Seventh Report of the International Committee on Taxonomy of Viruses.

[B4] Bielefeldt-Ohmann H, Meyer M, Fitzpatrick DR, Mackenzie JS (2001). Dengue virus binding to human leukocyte cell lines: receptor usage differs between cell types and virus strains. Virus Res.

[B5] Hung JJ, Hsieh MT, Young MJ, Kao CL, King CC, Chang W (2004). An external loop region of domain III of dengue virus type 2 envelope protein is involved in serotype-specific binding to mosquito but not mammalian cells. J Virol.

[B6] Martinez-Barragan JJ, del Angel RM (2001). Identification of a putative coreceptor on Vero cells that participates in dengue 4 virus infection. J Virol.

[B7] Moreno-Altamirano MM, Sanchez-Garcia FJ, Munoz ML (2002). Non Fc receptor-mediated infection of human macrophages by dengue virus serotype 2. J Gen Virol.

[B8] Ramos-Castaneda J, Imbert JL, Barron BL, Ramos C (1997). A 65-kDa trypsin-sensible membrane cell protein as a possible receptor for dengue virus in cultured neuroblastoma cells. J Neurovirol.

[B9] Reyes-del Valle J, del Angel RM (2004). Isolation of putative dengue virus receptor molecules by affinity chromatography using a recombinant E protein ligand. J Virol Methods.

[B10] Salas-Benito JS, del Angel RM (1997). Identification of two surface proteins from C6/36 cells that bind dengue type 4 virus. J Virol.

[B11] Yazi Mendoza M, Salas-Benito JS, Lanz-Mendoza H, Hernandez-Martinez S, del Angel RM (2002). A putative receptor for dengue virus in mosquito tissues: localization of a 45-kDa glycoprotein. Am J Trop Med Hyg.

[B12] Chen Y, Maguire T, Hileman RE, Fromm JR, Esko JD, Linhardt RJ, Marks RM (1997). Dengue virus infectivity depends on envelope protein binding to target cell heparan sulfate. Nat Med.

[B13] Tassaneetrithep B, Burgess TH, Granelli-Piperno A, Trumpfheller C, Finke J, Sun W, Eller MA, Pattanapanyasat K, Sarasombath S, Birx DL, Steinman RM, Schlesinger S, Marovich MA (2003). DC-SIGN (CD209) mediates dengue virus infection of human dendritic cells. J Exp Med.

[B14] Jindadamrongwech S, Smith DR (2004). Virus Overlay Protein Binding Assay (VOPBA) reveals serotype specific heterogeneity of dengue virus binding proteins on HepG2 human liver cells. Intervirology.

[B15] Jindadamrongwech S, Thepparit C, Smith DR (2004). Identification of GRP 78 (BiP) as a liver cell expressed receptor element for dengue virus serotype 2. Arch Virol.

[B16] Thepparit C, Smith DR (2004). Serotype-specific entry of dengue virus into liver cells: identification of the 37-kilodalton/67-kilodalton high-affinity laminin receptor as a dengue virus serotype 1 receptor. J Virol.

[B17] Cao W, Henry MD, Borrow P, Yamada H, Elder JH, Ravkov EV, Nichol ST, Compans RW, Campbell KP, Oldstone MB (1998). Identification of alpha-dystroglycan as a receptor for lymphocytic choriomeningitis virus and Lassa fever virus. Science.

[B18] Borrow P, Oldstone MB (1992). Characterization of lymphocytic choriomeningitis virus-binding protein(s): a candidate cellular receptor for the virus. J Virol.

[B19] Nordhoff E, Egelhofer V, Giavalisco P, Eickhoff H, Horn M, Przewieslik T, Theiss D, Schneider U, Lehrach H, Gobom J (2001). Large-gel two-dimensional electrophoresis-matrix assisted laser desorption/ionization-time of flight-mass spectrometry: an analytical challenge for studying complex protein mixtures. Electrophoresis.

[B20] Egelhofer V, Bussow K, Luebbert C, Lehrach H, Nordhoff E (2000). Improvements in protein identification by MALDI-TOF-MS peptide mapping. Anal Chem.

[B21] Falconar AK (1999). Identification of an epitope on the dengue virus membrane (M) protein defined by cross-protective monoclonal antibodies: design of an improved epitope sequence based on common determinants present in both envelope (E and M) proteins. Arch Virol.

[B22] Perkins DN, Pappin DJ, Creasy DM, Cottrell JS (1999). Probability-based protein identification by searching sequence databases using mass spectrometry data. Electrophoresis.

[B23] de Hoog CL, Foster LJ, Mann M (2004). RNA and RNA binding proteins participate in early stages of cell spreading through spreading initiation centers. Cell.

[B24] Wang KS, Kuhn RJ, Strauss EG, Ou S, Strauss JH (1992). High-affinity laminin receptor is a receptor for Sindbis virus in mammalian cells. J Virol.

[B25] Ludwig GV, Kondig JP, Smith JF (1996). A putative receptor for Venezuelan equine encephalitis virus from mosquito cells. J Virol.

[B26] Protopopova EV, Konavalova SN, Loktev VB (1997). [Isolation of a cellular receptor for tick-borne encephalitis virus using anti-idiotypic antibodies]. Vopr Virusol.

[B27] Donaldson EA, McKenna DJ, McMullen CB, Scott WN, Stitt AW, Nelson J (2000). The expression of membrane-associated 67-kDa laminin receptor (67LR) is modulated in vitro by cell-contact inhibition. Mol Cell Biol Res Commun.

[B28] Evander M, Frazer IH, Payne E, Qi YM, Hengst K, McMillan NA (1997). Identification of the alpha6 integrin as a candidate receptor for papillomaviruses. J Virol.

[B29] Gavrilovskaya IN, Brown EJ, Ginsberg MH, Mackow ER (1999). Cellular entry of hantaviruses which cause hemorrhagic fever with renal syndrome is mediated by beta3 integrins. J Virol.

[B30] Bergelson JM, Chan BM, Finberg RW, Hemler ME (1993). The integrin VLA-2 binds echovirus 1 and extracellular matrix ligands by different mechanisms. J Clin Invest.

[B31] Bergelson JM, Shepley MP, Chan BM, Hemler ME, Finberg RW (1992). Identification of the integrin VLA-2 as a receptor for echovirus 1. Science.

[B32] Weigel-Kelley KA, Yoder MC, Srivastava A (2003). Alpha5beta1 integrin as a cellular coreceptor for human parvovirus B19: requirement of functional activation of beta1 integrin for viral entry. Blood.

[B33] Graham KL, Zeng W, Takada Y, Jackson DC, Coulson BS (2004). Effects on rotavirus cell binding and infection of monomeric and polymeric peptides containing alpha2beta1 and alphaxbeta2 integrin ligand sequences. J Virol.

[B34] Chu JJ, Ng ML (2004). Interaction of West Nile virus with alpha v beta 3 integrin mediates virus entry into cells. J Biol Chem.

[B35] Chiu CY, Mathias P, Nemerow GR, Stewart PL (1999). Structure of adenovirus complexed with its internalization receptor, alphavbeta5 integrin. J Virol.

[B36] Kessels MM, Engqvist-Goldstein AE, Drubin DG, Qualmann B (2001). Mammalian Abp1, a signal-responsive F-actin-binding protein, links the actin cytoskeleton to endocytosis via the GTPase dynamin. J Cell Biol.

[B37] Kay-Jackson PC, Goatley LC, Cox L, Miskin JE, Parkhouse RM, Wienands J, Dixon LK (2004). The CD2v protein of African swine fever virus interacts with the actin-binding adaptor protein SH3P7. J Gen Virol.

[B38] Moir RD, Yoon M, Khuon S, Goldman RD (2000). Nuclear lamins A and B1: different pathways of assembly during nuclear envelope formation in living cells. J Cell Biol.

[B39] Goldman RD, Gruenbaum Y, Moir RD, Shumaker DK, Spann TP (2002). Nuclear lamins: building blocks of nuclear architecture. Genes Dev.

[B40] Kondo H, Rabouille C, Newman R, Levine TP, Pappin D, Freemont P, Warren G (1997). p47 is a cofactor for p97-mediated membrane fusion. Nature.

[B41] Cardosa MJ, Wang SM, Sum MS, Tio PH (2002). Antibodies against prM protein distinguish between previous infection with dengue and Japanese encephalitis viruses. BMC Microbiol.

