# Supplementary figures and images for: Two dimensional VOPBA reveals laminin receptor (LAMR1) interaction with dengue virus serotypes 1, 2 and 3
Source: Virol J. 2005 Mar 25;2:25. doi: 10.1186/1743-422X-2-25 (PMC1079963; doi:10.1186/1743-422X-2-25)

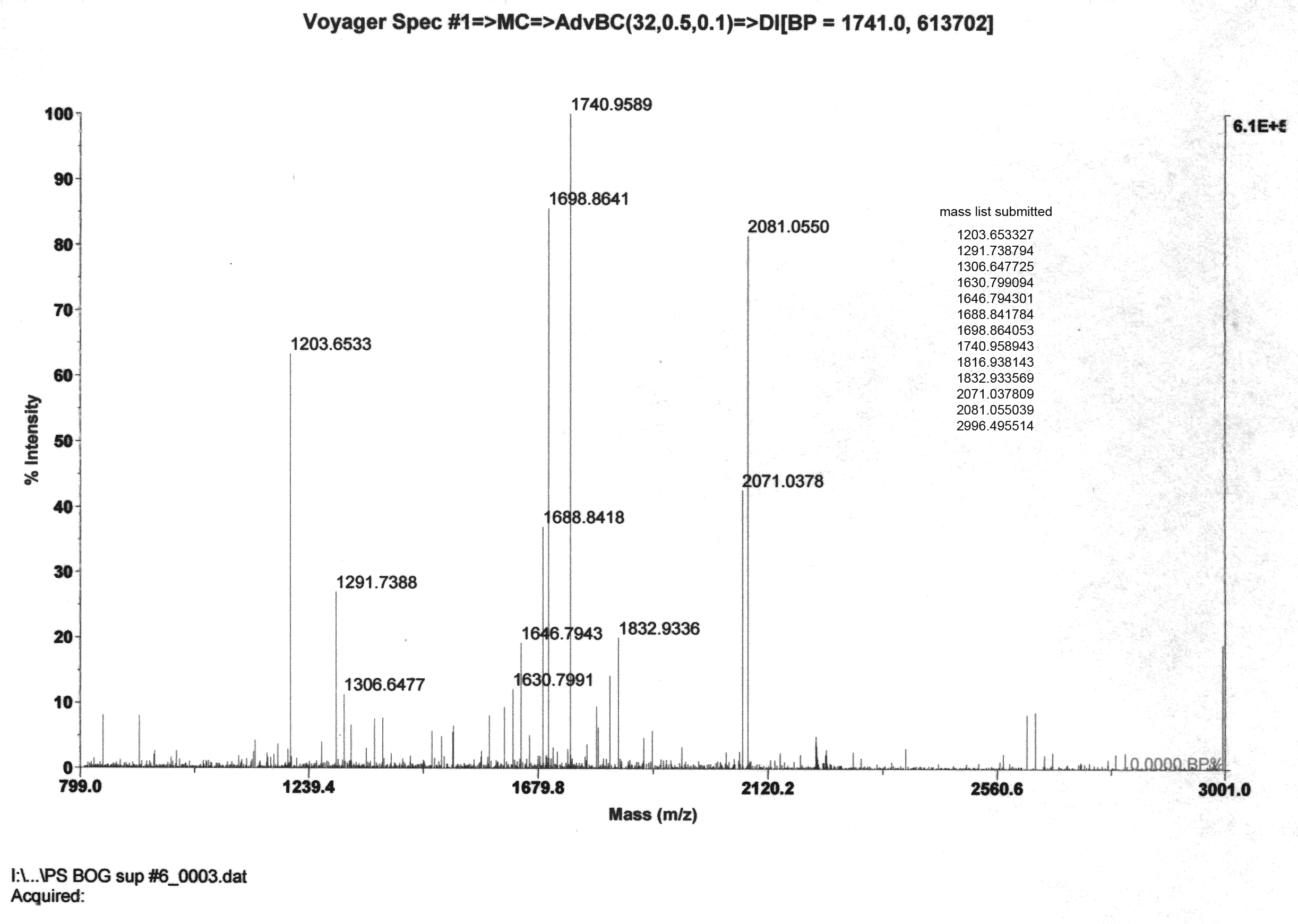

Supplement: Additional File 1 — Spectra acquired with mass list submitted for search Image of spectra obtained by MALDI-TOF for the spot from the 2D gel of βOG extracts identified as LAMR1. [file 1743-422X-2-25-S1.tiff]

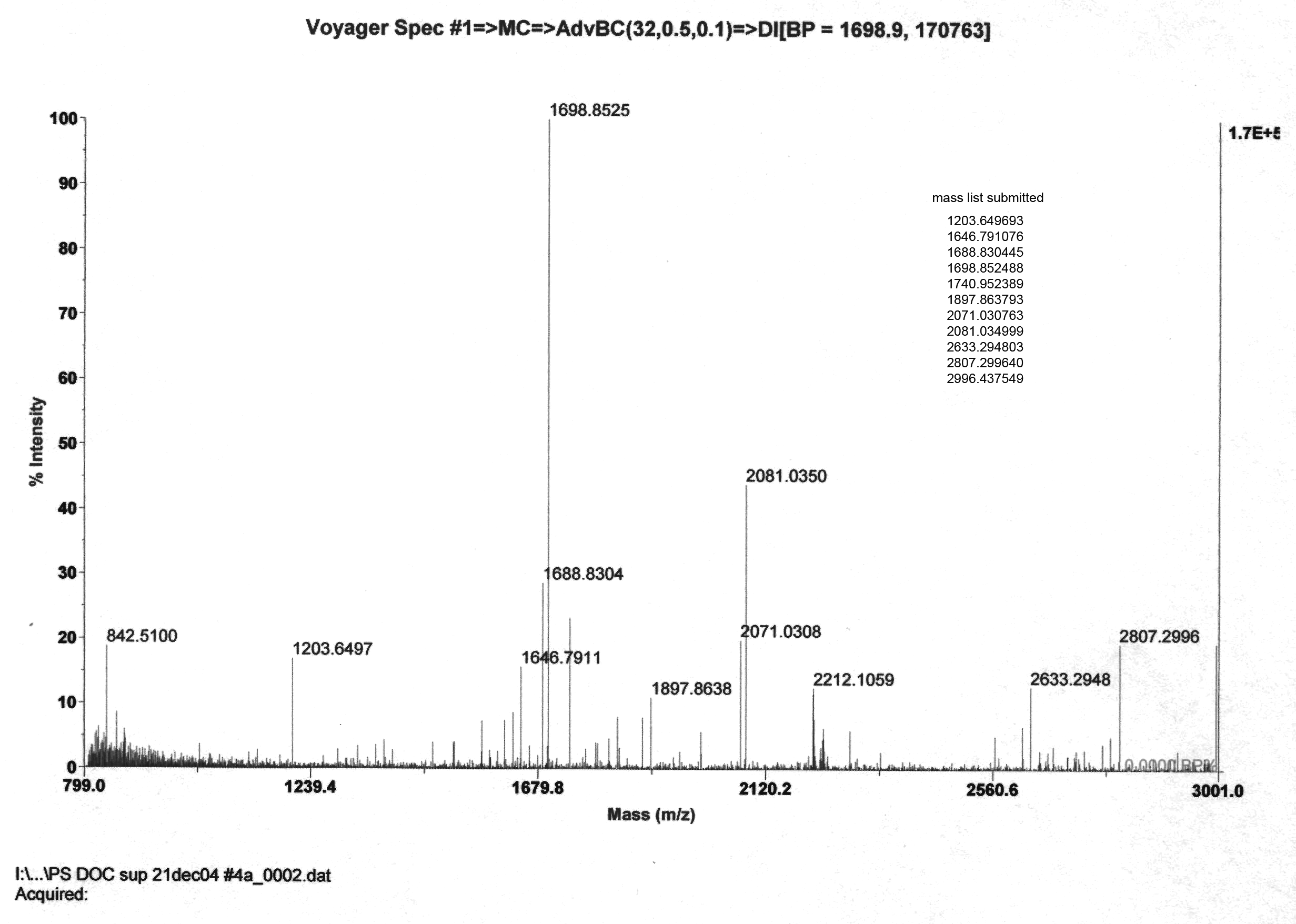

Supplement: Additional File 2 — Spectra acquired with mass list submitted for search Image of spectra obtained by MALDI-TOF for the spot from the 2D gel of NaDOC extracts identified as LAMR1. [file 1743-422X-2-25-S2.tiff]

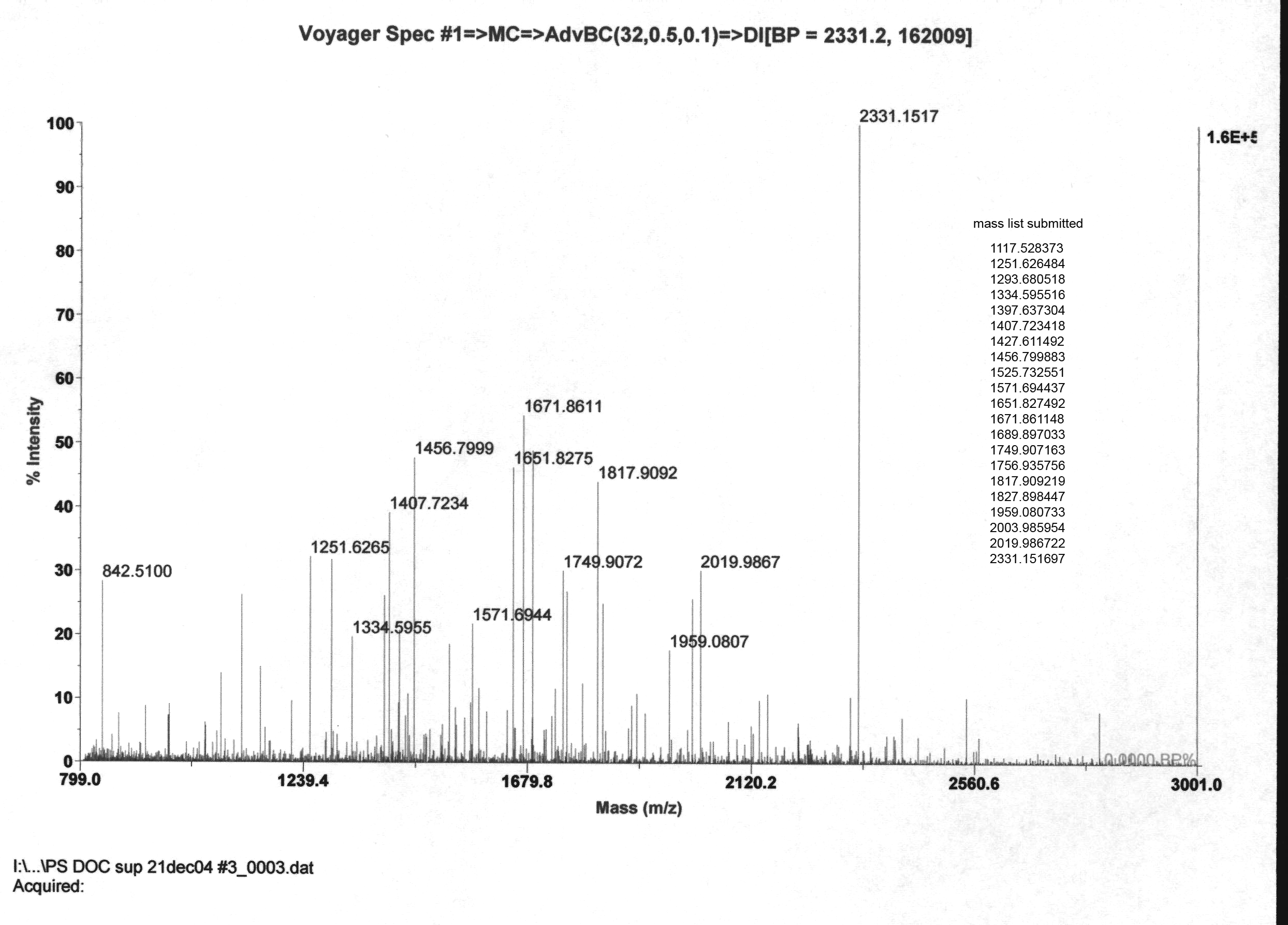

Supplement: Additional File 3 — Spectra acquired with mass list submitted for search Image of spectra obtained by MALDI-TOF for the spot identified as lamin B1. [file 1743-422X-2-25-S3.tiff]

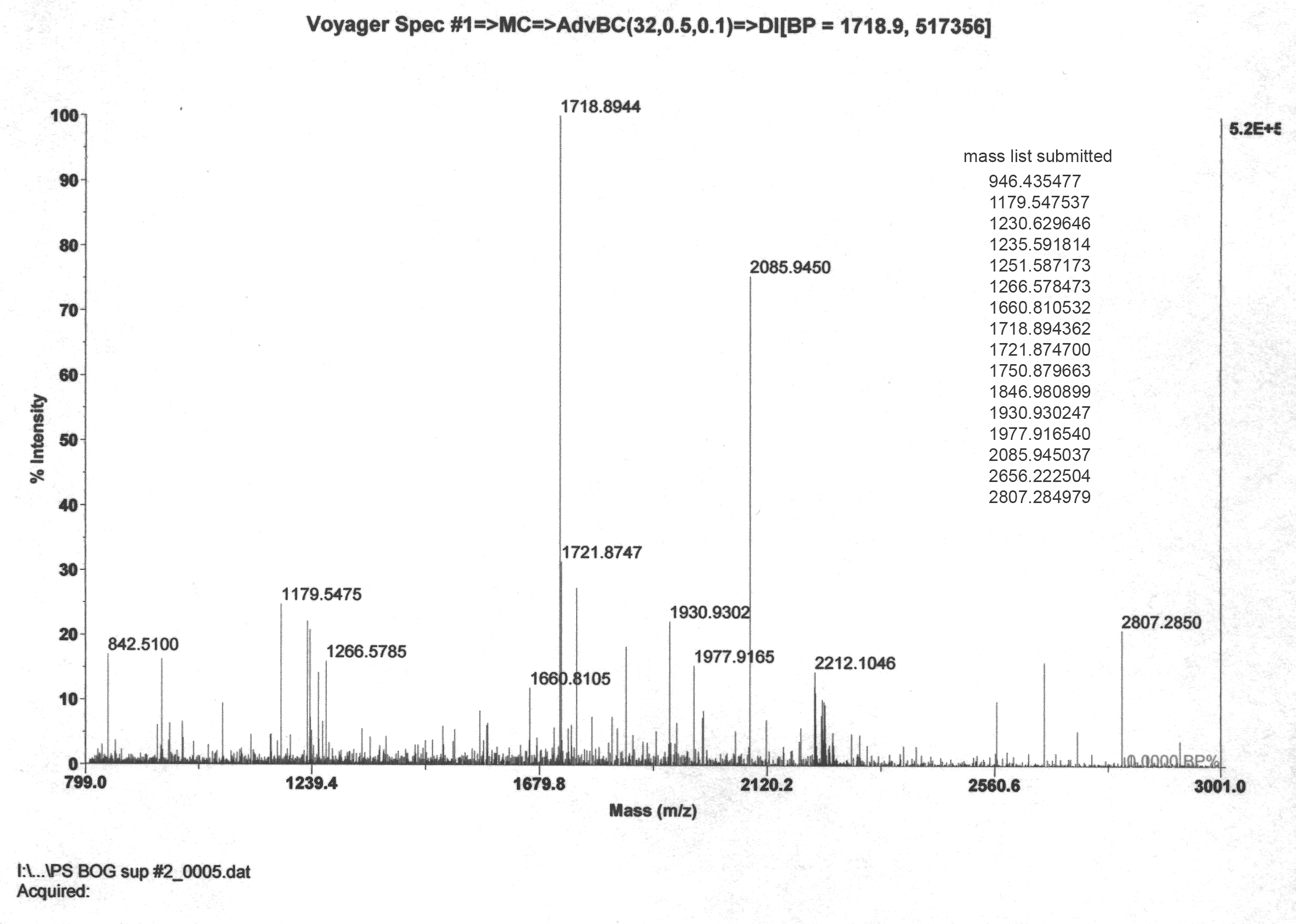

Supplement: Additional File 4 — Spectra acquired with mass list submitted for search Image of spectra obtained by MALDI-TOF for the spot identified as Hip 55. [file 1743-422X-2-25-S4.tiff]

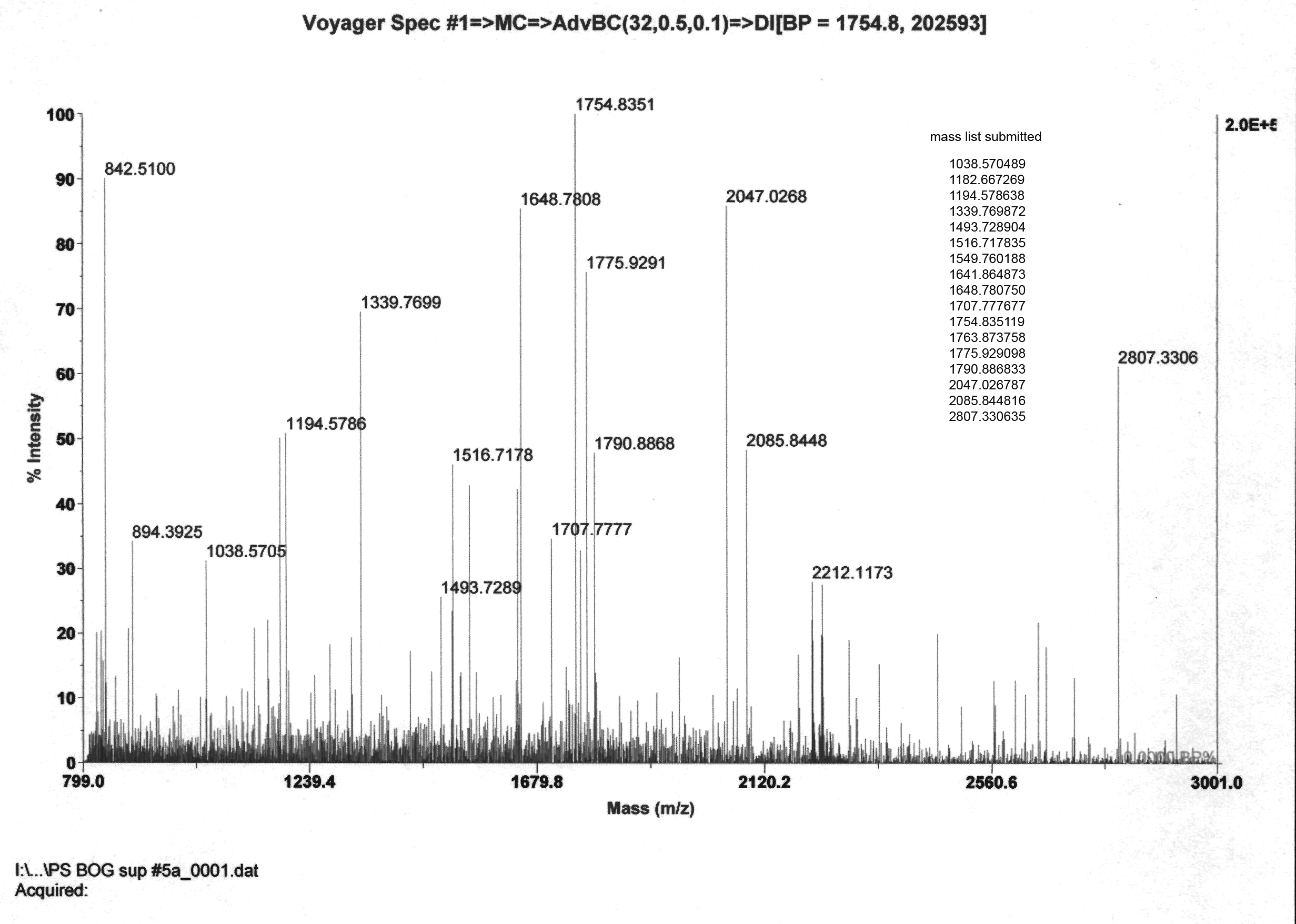

Supplement: Additional File 5 — Spectra acquired with mass list submitted for search Image of spectra obtained by MALDI-TOF for the spot identified as p47 protein. [file 1743-422X-2-25-S5.tiff]
